# Expression of bovine interleukin 15 and evaluation of its biological activity *in vitro*

**DOI:** 10.14202/vetworld.2015.295-300

**Published:** 2015-03-12

**Authors:** N. Vijay, J. Lijo, H. J. Dechamma, V. Bhanuprakash, B. Suresh, K. Ganesh, G. R. Reddy

**Affiliations:** 1 FMD Research Laboratory, Indian Veterinary Research Institute, Hebbal, Bangalore, Karnataka, India; 2 FMD QC/QA Lab, Indian Veterinary Research Institute, Hebbal, Bangalore, Karnataka, India; 3 FMD Vaccine Production Unit, Indian Veterinary Research Institute, Hebbal, Bangalore, Karnataka, India

**Keywords:** Bovine interleukin 15, Carnitine Palmitoyl Transferase 1a, Memory CD8^+^T cells

## Abstract

**Background/Aim:**

Recent studies have shown that interleukin-15 (IL-15)is a critical factor for the development and proliferation of CD8^+^ memory T cells. The aim of present study is to study the role bovine IL-15 (bIL-15)in activation pathway of bovine CD8^+^ T cells if any, which will be useful in designing the adjuvant to increase the duration of immunity of the vaccine preparations.

**Materials and Methods:**

Coding region of bIL-15 (489) was amplified from cDNA of lipopolysaccharide-induced bovine peripheral blood mononuclear cells (PBMCs) using gene specific primers and cloned into pcDNA3.1^+^. Mature length of bIL-15 was amplified using gene specific primers and cloned into pET32a for expression studies. Expressed fusion protein was purified using Ni-Nitrilotriacetic acid agarose affinity chromatography and analyzed by SDS-Polyacryamide gel electrophoresis (PAGE) and western blotting. Biological activity of purified protein was analyzed by quantitative Polymerase Chain Reaction (qPCR) for an increase in levels of Bcl2, STAT3 and STAT5a using cDNA synthesized from RNA of PBMCs induced with different concentrations of purified bIL-15. Role of IL-15 in inducing memory CD8^+^ T cells was analyzed by qPCR for increase in the level of Carnitine Palmitoyl Transferase 1a (CPT1a) using cDNA synthesized from RNA of PBMCs induced with different concentrations of purified bIL-15.

**Results:**

Bovine IL-15 was amplified and analyzed by agarose gel electrophoresis, which showed a specific product of ~490bp, mature sequence was amplified using full-length as a template to get a product of ~350bp. The protein was expressed, purified and analyzed by SDS-PAGE and Western blotting, which showed a specific product of 32kDa. Biological activity of purified bIL-15 fusion protein showed an increase in levels of Bcl2, STAT3 and STAT5a with 5 fold, 9 fold, and 10 fold increases as analyzed by qPCR, respectively. Role of IL-15 in inducing memory T cells showed an increase in expression level of CPT1a at 2.5 fold increase as compared to control cells.

**Conclusion:**

Bovine IL-15 has been successfully cloned and expressed in our work, and the biological activity shows that the purified fusion protein is biologically active. As there is an increase in levels of CPT1a an enzyme critical for survival of memory T cells, IL-15 can be used for increase in the memory response, which can be used as an adjuvant with viral vaccines for increasing the immunity.

## Introduction

Interleukin (IL-15), a critical factor for the development, proliferation and activation of effector, natural killer and CD8^+^ memory T cells, was first identified based on its ability to stimulate the IL-2 dependent CTLL-2 T cell lines in the presence of neutralizing IL-2 antibodies [1]. Mature IL-15 is a glycoprotein of 14-15 kDa in length with 2 inter disulfide bonds, carries out its signal transduction by reacting with specific receptors IL-15 Rαβγ, where in it shares a common receptor βγc with IL-2 and Rα is specific for IL-15. As it shares a common receptor of IL-2 (IL-2 βγc), it delivers its action through the activation of Jak/STAT pathway similar to IL-2 [2,3]. Specific activity of IL-15 is attributed to another receptor IL-15Rα, through which it opposes the activity of IL-2, by inducing Bcl2, which is an anti-apoptotic factor [4].

Conversion of effector T cells to memory T cells after an infection is a critical process, which is important to combat the re infection of the same pathogen. Generation of memory T cells is a complex process wherein there will be changes in cellular processes, mainly the shift of metabolism from glycolysis, which is prominent in effector cells [5,6] to oxidative phosphorylation, which is also a major metabolic pathway in case of naïve T cells for their survival and maintenance. Switch of metabolism has been clearly shown during the activation of the naïve T cells [7-9]. With these reports, it is clear that the switch of metabolism between naïve, effector and memory cells from oxidative phosphorylation to glycolysis and back to oxidative phosphorylation is a major process to decide the T cell fate. Albeit there is involvement of some cytokines for survival and maintenance of resting T cells, and how the change in metabolism from glycolysis to oxidative phosphorylation during development of memory T cell is less understood.

Studies have shown that the modulation of fatty acid oxidation (FAO) *in vitro* increased in CD8^+^ memory T cells development after vaccination [10]. The pathway involved in the generation of CD8^+^ memory T cells where in the switch of metabolic profile and taking place has been discovered by Van der windt *et al*. [11] showing the involvement of IL-15 in converting effector CD8^+^ T cells to memory CD8^+^ T cells and its role in switching metabolism from glycolysis into oxidative phosphorylation by utilizing FAO. IL-15 increases the rate of FAO in turn facilitating oxidative phosphorylation by increasing the expression of Carnitine palmitoyl transferase 1a (CPT1a), a major enzyme, which catalyzes the first step of FAO.

In the present study, we report the expression of the biologically active IL-15 in *Escherichia coli*, and its role in inducing Cpt1a enzyme, a critical step in converting effector T cells to memory T cells.

## Materials and Methods

### Ethical approval

All the procedures were carried out with prior ethical permission from the concerned authority (Institute Biosafety Committee), and the prior approval was taken from IAEC (Institute Animal Ethical Committee-Ref N. F.8-56-Vol.II/RCSS/2014-2015).

### Cloning and expression of bovine IL-15 (bIL-15)

Peripheral blood was collected from *Bos indicus* maintained in Yelahanka animal facility, IVRI, Bengaluru, for isolating peripheral blood mononuclear cells (PBMCs). PBMCs were isolated using Histopaque 1077. Coding region of bIL-15 (489) was amplified from cDNA of LPS-induced bovine PBMCs using gene specific primers with RE sites for *Bam* HI and *Xho* I for forward and reverse primers, respectively. Amplified gene was digested with *Bam* HI and *Xho* I and ligated with pcDNA 3.1 previously digested with the enzymes *Bam* HI and *Xho* I. Ligation mix was transformed into Top10 competent cells and the colonies were screened for the recombinants by performing colony polymerase chain reaction (PCR) using bIL-15 gene specific primers. Plasmids isolated from Positive colonies were analyzed by restriction enzyme digestion. The confirmed recombinants were sequenced.

Primers list:

- IL-15 Forward: 5’GGCGGATCCATGAGAA TTTTGAAACCATATTT 3’
- IL-15 Reverse: 5’GGCGCTCGAGTCAAGAAG TGTTGATGAACAT 3’
- IL-15 Mature: 5’GCGGATCCAACTGGCAGT ATGTAATAAATG

Mature sequence of bIL-15 was amplified using primers to remove the leader peptide and for getting authentic N terminal peptide using full-length gene as a template. Amplified mature gene was cloned in pET32a for expression in *E. coli* cells. Optimum level of expression was checked by expressing the protein at different concentrations of IPTG (0.5 mM, to 1.5 mM) and at different time intervals (2 h, 3 h, 4 h and 5 h). Expressed protein was analyzed by SDS Polyacryamide gel electrophoresis (PAGE). Recombinant protein was purified in denatured conditions using Ni-Nitrilotriacetic acid (NTA) affinity chromatography and analyzed by SDS PAGE.

### Biological activity of recombinant IL-15

PBMCs were isolated from the bovine blood using Histopaque 1077. Final concentrations of cells were made to 10^6^ cells/ml using RPMI with 10% serum. The cells were dispensed into 6 well plates at equal concentrations and the same were incubated with different concentrations (500 ng, 1 μg) of recombinant bIL-15 (rbIL-15). Cells were harvested and with Trizol after 12 h of incubation. RNA was isolated by following the standard protocol. cDNA was synthesized from the purified RNA using Oligo dT primer using MMLV RT. qPCR was carried out for checking the levels of Bcl2, STAT3, and STAT5 using the bovine specific real time primers designed using primer select software of NCBI. The 2^−∆∆Ct^ values will be calculated as per the method [12] taking GAPDH as a housekeeping gene.

Primers list:

- Bcl2 forward: 5’ AGCGTCAACCGGGAGAT GT 3’
- Bcl2 Reverse: 5’ TAGGGCCATACAGCTCC AC 3’
- Stat5A Forward: 5’ GTACCCACAGAACCCTG AC 3’
- Stat5A Reverse: 5’ AGAGAGGGCTCCAGACT GT 3’
- Stat3 Forward: 5’ CAATACCATTGACCAGCCG AT 3’
- Stat3 Reverse: 5’ GAGCGACTCAAACTGCC CT 3’

### Induction of Cpt1a

Cpt1a is an important enzyme in the path way that induces the conversion of effector CD8^+^ T cells to CD8^+^ T memory cells [11]. Induction of the enzyme is the marker for the development of CD8^+^ T memory cells. We evaluated the role of bIL-15 in the induction of Cpt1a in the PBMCs. The cells were incubated with different concentrations (500 ng, 1 μg) of rbIL-15 fusion protein. Cells were harvested and lysed with Trizol after 12 h of incubation. RNA was isolated by following the standard protocol. cDNA was synthesized from the purified RNA using Oligo dT primer using MMLV RT. qPCR was carried out for checking the levels of Cpt1a using the bovine specific real time primers designed using primer select software of NCBI.

- Cpt1a Forward: 5’ CCGGGAGGAAATCAA ACCGA 3’
- Cpt1a Reverse: 5’ TCCCGGGATCCGAGAA GTAT 3’

## Results

### Cloning and expression of bIL-15

**Figure-1:**
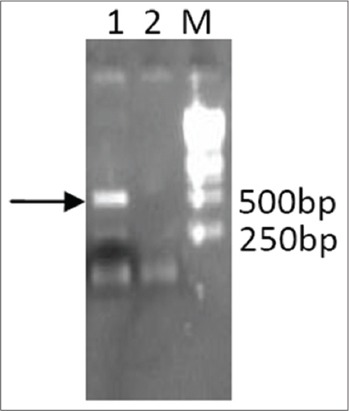
cDNA synthesized from RNA purified from the LPS-induced bovine lymphocytes was used as a template for the amplification of IL-15. The amplified product was analyzed using 1.2% agarose gel electrophoresis. Lane 1: Arrow showing the amplified product of bIL-15 of length ~500 bp, Lane M: Gene ruler 1 kb DNA ladder (Fermentas SM0314).

**Figure-2:**
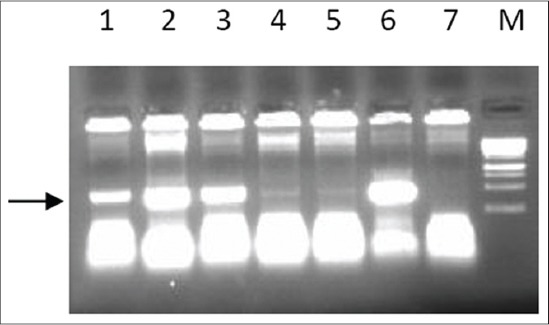
The suspected colonies were subjected to colony PCR with IL-15 bovine specific primers. The amplified products were analyzed by 1.2% agarose gel electrophoresis. Lane 1, 2, 3 and 6: Positive clones showing amplified product of length ~500 bp, Lane 4, 5 and 7: Negative clones showing no specific bands, Lane M: Gene ruler 1 kb DNA ladder (Fermentas SM0314).

**Figure-3:**
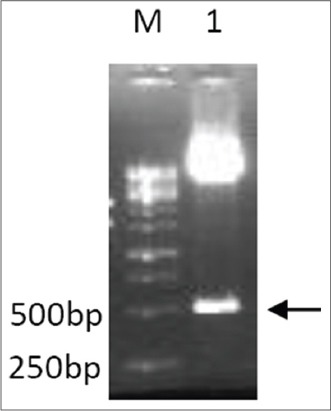
The purified bIL-15/pcDNA plasmid was subjected to Restriction digestion with *Bam* HI and *Xho* I to release the fragment. The reaction mix was analyzed by 1.2% agarose gel electrophoresis. Lane M: Gene ruler 1 kb DNA ladder (Fermentas SM0314), Lane 1: Arrow showing a released fragment of length ~500 bp.

Sequence coding for bIL-15 was amplified by reverse transcription PCR of the RNA extracted from the activated lymphocytes. The PCR products were the confirmed by agarose gel electrophoresis (Figure-1). Lane 1 of Figure-1 showing the band of 500 bp corresponds to bIL-15 coding sequence. The purified PCR products were digested and ligated with similarly digested pcDNA3.1 vector, and transformed colonies were screened by Colony PCR. Specific band of 500 bp seen in the gel (lane 1, 2, 3 and 6 Figure-2), corresponds to the recombinants with bIL-15 insert. The plasmids isolated from the recombinant clones subjected to restriction digestion analysis (Lane 1 Figure-3) showed the release of the fragment of 500 bp in size.

**Figure-4:**
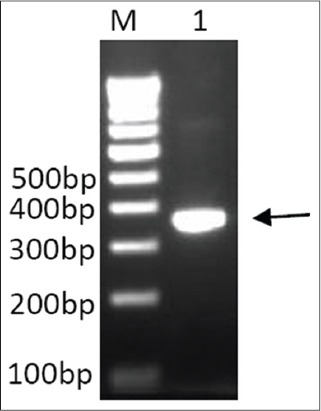
Full-length bIL-15 was used as a template to amplify the mature bIL-15 using mature IL-15 forward and reverse primer. Lane M: Gene ruler 1 Kb DNA ladder (Fermentas SM0314) Lane 1: Arrow showing amplified mature bIL-15 of length ~350bp.

**Figure-5:**
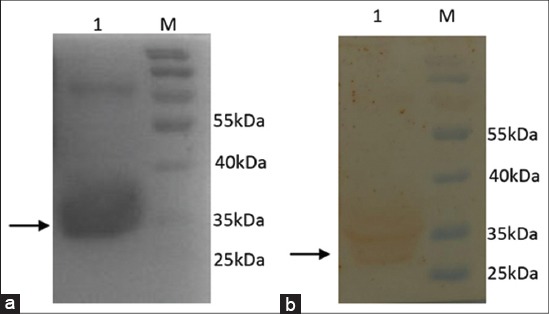
Analysis of *Escherichia coli* expressed bIL-15 mature fusion protein. (a) The lysate of BL21 (DE3) expressing bIL-15 fusion protein was subjected to Ni-Nitrilotriacetic acid affinity chromatography. Purified protein was analyzed by 12% SDS-PAGE, Lane 1: Showing purified bIL-15 fusion protein, Lane M: Page ruler pre-stained protein ladder (Pierce 26616), (b) The protein analyzed by SDS-PAGE was subjected to Western Blotting. The protein bands were transferred to Nitrocellulose membrane and developed using anti-His tag antibodies and ODD as substrate, Lane 1: Showing purified bIL-15 fusion protein, Lane M: Page ruler pre-stained protein ladder (Pierce 26616).

Mature sequence was amplified using full-length pcDNA plasmid as a template, which is found to be of the size ~350 bp (lane 1, Figure-4). Amplified mature bIL-15 sequence was cloned in pET32a and transformed into BL21 cells and recombinants were screened by colony PCR and confirmed by restriction digestion. To optimize the expression, three colonies were selected for expression at different concentrations of IPTG (0.5-1.5 mM) and samples collected at different time intervals (1-5 h) using different concentrations of IPTG (0.5-1.5 mM). The optimal conditions found to be 1 mM IPTG and 5 h induction. These conditions were used for further induction. The expressed proteins were characterized by 12% gel SDS PAGE. The expressed bIL-15 was purified using Ni-NTA affinity chromatography in denaturing conditions using 8M Urea. The eluted protein was analyzed by SDS PAGE, which showed a band corresponding to 32 kDa as expected (Lane 1, Figure-5a). The specificity of the protein was confirmed by Western blotting using anti-human IL-15 antibody (Biolegend), which showed the band corresponding to IL-15 (Lane 1, Figure-5b).

### Evaluation of biological activity

**Figure-6:**
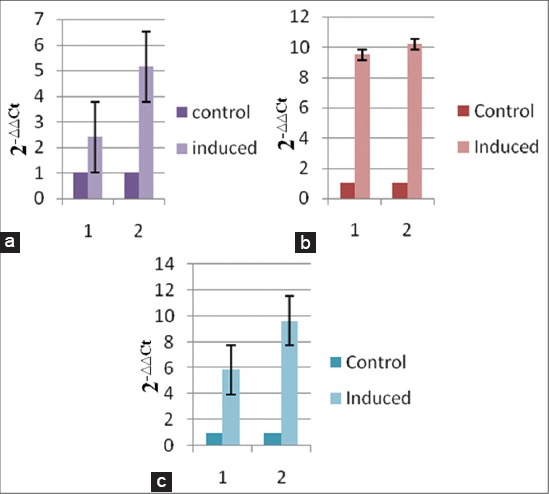
Bovine PBMCs was isolated from peripheral blood, incubated with 500 ng and 1 μg of purified bIL-15 fusion protein for 12 h. cDNA synthesized from the RNA of bIL-15-induced PBMCs was used as template for analyzing the levels of Bcl2 (a), Stat3 (b) and Stat5 (c) by qPCR using gene specific primers (Note: Y-axis: Fold increase in expression, X-axis: different concentrations of bIL-15 fusion protein, 1-500ng and 2-1 µg).

Biological activity of bIL-15 was measured by its stimulation of anti-apoptotic factor Bcl2 and signal transduction molecules STAT 3 and STAT5 by qRTPCR. Bovine PBMCs were stimulated with different concentrations of purified IL15 (500 ng and 1μg). mRNAs coding for different ISGs were quantified by qRTPCR by relative quantification method. The 2^−∆∆CT^ values were calculated as described earlier. 1μg of IL 15 was able to induce optimal levels of Bcl2 expression (Figure-6a). Bcl2 protein gene expression was increased by 5 folds. Since, STAT3 and STAT5 proteins are involved in cytokine signalling in JAK-STAT pathway, effect of IL-15 protein on the expression of STAT 3 and STAT5 was studied by Real Time PCR by quantifying the STAT3 and STAT5 coding mRNAs. The 2^−∆∆CT^ values (Figure-6b and c) showed that bIL15-induced the STAT 3 by 10 folds and STAT 5 by 9 folds as compared to the untreated cells.

### Activation of Cpt1a

**Figure-7:**
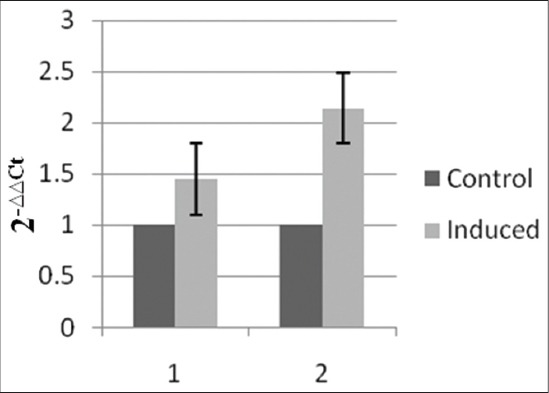
Bovine PBMCs was isolated from peripheral blood, incubated with 500ng and 1 μg of purified bIL-15 fusion protein for 12 hrs. cDNA synthesized from the RNA of bIL-15-induced PBMCs was used as template for analyzing the levels of Carnitine Palmitoyl Transferase 1a by qPCR using gene specific primers (Note: Y-axis: Fold increase in expression, X-axis: different concentrations of bIL-15 fusion protein, 1-500 ng and 2-1 µg).

Role of IL-15 on the induction of CPT1a, which is an important step for the conversion of activated CD8^+^T cells, to memory CD8^+^ T cells was evaluated *in vitro*. Effect of the expressed bIL-15 protein on the expression of Cpt1a was analyzed by quantifying the mRNAs coding for these molecules by qPCR using specific primers in the stimulated lymphocytes. Cpt1a which plays an important role in induction of CD8^+^ T memory cells was stimulated by more than 2 folds at 1 μg, while, it was 1.5 fold increase in the expression at 500 ng conc (Figure-7).

## Discussion

Cellular responses for the primary infection involve activation of naive T and B lymphocytes. Cytokines like IL-2 were shown to play an important role for this activation. Generation of memory T cells is an important step for long-lasting immune responses, wherein the small population of effector cells will get concerted into memory cells, which is influenced by cytokines *viz*., IL-15, IL-7, and IL-12. Studies have proved that the expression of IL-15 and IL-7 critical for the generation of memory CD 8^+^ T cells and their maintenance [13]. With this property of enhancing the immune cell proliferation and activation of CD8^+^ T memory cells, bIL-15 could be a good molecular adjuvant to enhance the immune responses, as well as the duration. In our study, we report the cloning and expression of bIL-15 in *E. coli*. The sequence data have shown *Bos indicus* IL-15 has a homology of 99% to the *Bos Taurus* IL-15. Primers designed to amplify the sequence coding for the mature peptide. The fusion protein of 33 kDa was purified by NI-NTA affinity chromatography confirmed by western blot was used for the activity studies.

IL-15 activates JAK-STAT pathway and *src* related tyrosine kinase pathways through which it modulates the cell proliferating activities. In JAK-STAT pathway, it activates the STAT3 and STAT5 molecules [2,3] and Bcl2 in the activation of *src* related tyrosine kinase pathway [4]. To evaluate the biological activity, we treated bovine PBMCs with purified IL-15 and the mRNA levels of STAT3, STAT5 and Bcl2 were quantitated by qPCR. Bcl2 protein gene expression was increased by 5 folds where as STAT3 and STAT5 ten and nine folds, respectively. These results are in agreement with the reported work by [2-4].

It has been established that cytokines are involved in each and every step of development and metabolism of immune cells. Variations in the metabolic status in different stages of immune cells have been identified, Naïve and memory T cells utilizes oxidative phosphorylation for their survival, wherein the effector cells utilizes glycolysis for their activity as sudden burst of energy is required for cells to perform their activity [5,14]. Studies on the metabolic profiles of memory CD8^+^ T cells revealed that, these cells utilize for their survival using the FAO as a source of energy inside the mitochondria [10,15,16]. Cpt1a is critical for the transport of fatty acids in to mitochondria to be utilized in the oxidative phosphorylation. It was shown that the stimulation needed for increased expression of Cpt1a will be provided by IL-15 [11,15]. To see the role of IL-15 in Cpt1a activation, we incubated bovine PBMCs with expressed IL-15 and the mRNA levels of Cpt1a was measured by qPCR. Two-fold increase was seen in Cpt1a mRNA levels in bIL-15 treated PBMCs indicating the stimulation of the Cpt1a expression. Further studies are needed to analyze the stimulation of memory T and B cells with IL-15 stimulation *in vitro* as well *in vivo*. The studies will provide the insights to analyze IL-15 as a molecular adjuvant to improve the immune responses and duration of immunity.

## Conclusion

The bIL-15 has been successfully cloned, expressed and purified. Biological activity assessed by stimulating bovine PBMCs showed that the purified protein is biologically active. PBMCs stimulated with purified protein also showed increased in expression of Cpt1a gene, which is a critical step in transforming effector CD8^+^ T cells into memory CD8^+^ T cells.

## Authors’ Contributions

The work was designed under the guidance of GRR, HJD, VB, BS and KG. The sample collection and processing was carried out by NV and LJ. Cloning, expression and real time analysis was done by NV and LJ under the supervision of GRR. Manuscript was prepared and revised by HJD, VB, BS and KG. All the authors have approved the final manuscript.
